# Mpox on *Reddit*: a Thematic Analysis of Online Posts on Mpox on a Social Media Platform among Key Populations

**DOI:** 10.1007/s11524-023-00773-4

**Published:** 2023-08-14

**Authors:** Chenglin Hong

**Affiliations:** Department of Social Welfare, UCLA Luskin School of Public Affairs, 3250 Public Affairs Building, Los Angeles, CA USA

**Keywords:** Mpox, GBMSM, Social media, Thematic analysis, Stigma, Vaccine

## Abstract

The 2022–2023 mpox outbreak has disproportionately impacted gay, bisexual, and other men who have sex with men (GBMSM). The US CDC recommended individuals to explore safer sexual practices that minimize the potential risk of exposure and also strongly encouraged for eligible individuals to prioritize vaccination. This study aimed to analyze social media data related to mpox on *Reddit* since the mpox outbreak and identify themes associated with the impact on social behaviors and social processes among targeted population. Publicly available data were collected from the social media *Reddit*. We extracted the summarized mpox-related posts since the beginning of May 2022 from popular subreddits that were popular among GBMSM. We thematically analyzed the content to identify the overall themes related to the GBMSM’s responses to the outbreak. There is an overall increase in the number of daily mpox-related posts, with three upticks in late May, late July, and early August 2022, which may correspond to the dates that the first mpox case was identified in the USA, the WHO declared a global public health emergency, and the US Department of Health and Human Services declared a public health emergency. Four themes were identified: (1) changes in sexual behaviors and social activities; (2) mpox vaccine attitude, uptake, and hesitancy; (3) perceived and experienced stigma and homophobia, and mental distress; and (4) online information-seeking and mutual aid and support. GBMSM changed their sexual behaviors and social activities to mitigate their exposure to the virus during this outbreak and actively sought and shared information about mpox vaccination in their respective settings, while some were hesitant due to concerns about side effects and potential effectiveness. Perceived and experienced stigma and discrimination on gay- and same sex-identify have impacted GBMSM’s mental health. Interventions to promote the mpox vaccine must address the historical medical mistrust and vaccine hesitancy among GBMSM.

## Introduction

The emerging mpox infection has been spreading rapidly around the globe and represents a new global health outbreak. Since early May 2022, over 88,000 confirmed cases have been identified in over 100 countries, and more than 93% of which were reported in countries that had not historically reported mpox at the time of writing this article [1]. For the second time in two years, the World Health Organization (WHO) declared the outbreak of a public health emergency and called for international collaboration and response to prevent the infection from further spreading [2]. In the USA, there have been over 30,000 mpox cases since the first confirmed patient on May 17, 2022, and the Department of Health and Human Services declared a public health emergency on August 4, 2022, given the increasing cases in multiple states including California and Illinois [3, 4]. While most of the confirmed patients experience mild to moderate symptoms, several mpox-related death have been reported in Brazil, Spain, Peru, and India [5]. Researchers also argue that, given many of the confirmed cases had no substantial travel history to mpox endemic regions, the virus might already be spreading in non-endemic countries through undetected transmission for an unknown period [6, 7], which increases the possibility of community spread as human transmission occurs due to physical, close, skin-to-skin contacts [1].

Yet, this outbreak predominantly affects gay, bisexual, and other men who have sex with men (GBMSM). In Spain, 93% of the cases reported having condomless sexual intercourse or having sex with multiple partners within the 3 weeks before the onset of symptoms was GBMSM [8]. A more comprehensive report documenting mpox infections across 16 countries suggested that sexual activity among gay and bisexual men was the most frequently suspected transmission route [9]. A more recent CDC report in the USA suggested that nearly all individuals with cases reported were cisgender and transgender men and 94% reported having sex or close intimate contact with another man during 3 weeks before symptom onset [10]. Furthermore, the outbreak has disproportionately impacted historically vulnerable populations such as GBMSM of color and people living with or at risk of HIV [11]. For instance, the percentage of reported cases among Black individuals in the USA had increased from 12 to 31% from May to July, 2022, and Black and Latino GBMSM accounted for more than half of confirmed cases [10]. A recent review of reported cases and pooled data from six outbreak clusters found that more than half (54.3%) were individuals living with HIV [12]. Similarly, in the USA, 41% of cases with available data were individuals with HIV infection [10]. There is also an argument that these disparities can be further exacerbated due to the greater challenges these populations face accessing mpox testing, treatment, and vaccination [13]. Urgent public health policy responses are needed to prevent this outbreak from becoming another pandemic and exacerbating the health disparities among the most vulnerable populations.

Lessons from previous disease outbreaks that predominately impact the GBMSM, such as HIV/AIDS and invasive meningococcal disease, as well as the recent COVID-19 pandemic, suggest that public health must maximize prevention efforts and work collaboratively with the community [14]. The historically complicated relationship between GBMSM and public health often presents additional barriers to preventive health service utilization. For instance, despite the scientific evidence on preventing HIV acquisition, the uptake of pre-exposure prophylaxis (PrEP) remains low among GBMSM due to reasons including medical mistrust and sexual orientation-based discrimination [15, 16]. Since the COVID-19 crisis, GBMSM and LGBTQ+ individuals also reported high vaccine hesitancy towards vaccine uptake due to concerns about vaccine safety, efficacy, and historically negative experiences with healthcare providers [17–19]. Lessons learned from the HIV/AIDS epidemic and the recent COVID-19 pandemic suggest that it is essential to understand the community’s perspectives in combating the mpox outbreak. Understanding GBMSM’s concerns and prioritizing their needs is critical for public health and policymakers to design, implement, and refine programs and interventions to monitor and control the potential crisis. In recent years, publicly available social media data, such as posts on *Twitter* and *Reddit*, have been used to describe health outcomes and predict health behaviors among key populations [20–24]. For example, several recent studies used publicly available comments and posts in specific subreddits to understand JUUL and e-cigarette use among Reddit users [22, 25]. Another recently published study also utilized Reddit posts in a popular subreddit for GBMSM to illustrate changes in discussion and general attitude towards HIV pre-exposure (PrEP) over time [21]. By analyzing publicly available posts on a social media platform, *Reddit*, this study aims to describe the characteristics and changes of mpox-related posts over time, explore the user-generation conversations and discussions on mpox, and synthesize the common themes of these discussions. The results will provide empirical data on how GBMSM mitigate their potential exposures and risks of mpox and will provide information for tailored mpox prevention interventions among GBMSM.

## Methods

### Study Design and Data Collection

This qualitative analysis and thematic synthesis utilized publicly available data on http://reddit.com (*Reddit*). Reddit is one of the most popular social media platforms around the globe, with 430 million active users and more than 100 thousand active communities [26]. All online communication and posts are organized into a “subreddit” by specific subjects and topics. Reddit has been used for research in the past especially among targeted populations and topics. For this analysis, data collection was conducted in several *subreddits that were popular among GBMSM*, including r/askgaybros, r/gay, r/bisexual, r/askgaymen, r/gaybros, and r/lgbt. These subreddits were selected by popularity, active subscribers, and based on previous studies and recommendations [21, 27–29]. For example, r/askgaybros is one of the largest *subreddit* communities with active discussions on various topics among gay, bisexual, and other sexual minority men. It describes itself as “where anyone can ask the manly men for their opinions on various topics”. In July 2023, more than 377,000 members subscribed to this *subreddit*.

Using Reddit and Pushchift.io’s application program interfaces (APIs), posts on these subreddits were mined through programs coded in Python. Both posts’ titles and body text were collected because the title only allows 300 characteristics, whereas body text has no limit on word counts, which contains more information. The dates of each post being posted were also extracted for further analysis to illustrate the change in the number of discussions over time. All posts were collected anonymously with no users’ information. After the data mined in six subreddits (including r/askgaybros, r/gay, r/bisexual, r/askgaymen, r/gaybros, and r/lgbt), all records were exported as CSV files, with each line representing a single post. Reddit posts submitted since the start of May through late September 2022 were collected as it corresponds to the timeline for the beginning of the recent outbreak. A combination of keywords, including “monkeypox”, “mpox”, and “pox”, was used to identify the posts with mpox-related content and discussions. All data related to this analysis were publicly available, and the study did not include any identifiable information or involve human interaction. Therefore, an ethical review approval was not required.

### Data Analysis and Synthesis

Search records from the six subreddits were imported into Rstudio for data processing and cleaning. First, duplicated posts were removed as some users may post the same content for discussion under different subreddits. Cleaned datasets were exported as Microsoft Excel files for qualitative coding and thematic analysis. All posts were analyzed thematically using an inductive coding approach [30]. Each post was read to generate initial codes and re-read with an identified code or codes applied to it. Each post was assessed for inclusion during the process, and posts unrelated to or not referred to as “mpox” were also removed, and this coding process was conducted by the leading author with the consultation of a senior researcher. Following Barbour’s framework, themes were identified as codes or collections of codes that consist of elements that represented a patterned concept [31]. Once identified, themes were organized into major themes and subthemes hierarchically, with major themes being those with a higher level of importance and significance. Posts were then organized according to descriptive headings, and overarching themes were identified. Of note, one unique post may contain multiple codes and themes. Descriptive statistics were used to present the number of codes and posts and changes over time. As suggested in previous research [32, 33], all the posts were altered and rephrased to reduce the risk of searchability of these posts to protect the privacy of Reddit users.

## Results

### Description of mpox-Related Posts on Reddit

**Fig. 1:**
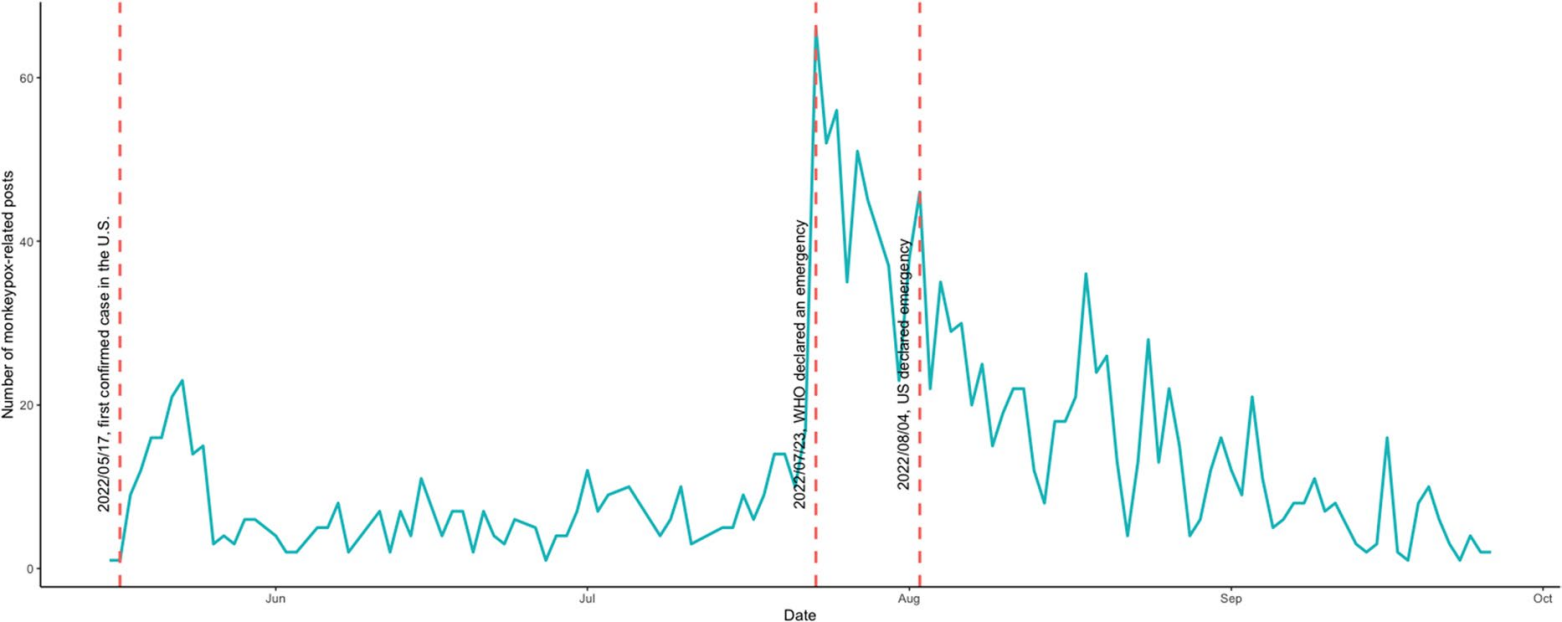
Trend of daily mpox-related posts on several subreddits since May 2022*. *Subreddits including r/askgaybros, r/gay, r/bisexual, r/askgaymen, r/gaybros, and r/lgbt

A total of 809 records were identified through the initial extracting process. After removing duplicates (some users would post the same topic under different subreddits, etc.) and irrelevant posts, *n* = 755 were included in the final analysis and synthesis. As presented in Fig. 1, there is an overall increasing trend of mpox-related posts in the selected subreddits, and there are three upticks in mid-May, late July, and the start of August 2022. The subreddit with the largest number of mpox-related posts was r/askgaybros (*n* = 452, 59.9%).

### Emerging Themes of Reddit Posts on mpox

Thematic analyses revealed four intersecting and overachieving themes: (1) changes in sexual behaviors and social activities; (2) mpox vaccine attitude, uptake, and hesitancy; (3) online information-seeking and mutual aid and support; and (4) perceived and experienced stigma and homophobia and mental distress. The numbers of posts related to the themes and example posts are presented in Table 1.

**Table 1:** Prevalence of themes emerged from mpox-related Reddit posts (*n* = 755)

Code description	*n*^*1*^	Example post^*2*^
Changes in sexual behaviors and social activities	*n = 151*	“I have kept myself from hooking up because of mpox. I haven’t been sleeping around in a while…” “I wanted to be safe, so I am not into dating someone or hooking up right now, given the COVID and monkeypox situation…” “No, I won’t be attending the bathhouse. Being potentially exposed the pox? No thanks...”
Mpox vaccine attitude, uptake, and hesitancy	*n = 179*	“I think I am at high risk than a lot of people. I want to be vaccinated…” “Got mine last week. Felt totally fine Friday but started to have some side effect on Saturday, I was sore all over and had a splitting headache that lasted all weekend.” “I am so grateful for the opportunity to get vaccinated. Thanks for the public health staff at the city of XXX…” “Does anyone know if there are several side efforts of the vaccine and any potential risks?”
Online information-seeking and mutual aid and support	*n = 219*	“I have a bf and we are open. Both of us are on PrEP. Are we eligible for the vaccine?” “How do you find your sexual partners? Are you still going to the bars and clubs?” “It seems like the Public Health is offering sexually active gay men to get vaccinated against mpox. And given the case numbers are rising. Will you have the vaccine if it was available in your area?
Perceived and experienced stigma and homophobia and mental distress	*n = 158*	“Anyone else getting tired of being blamed for HIV and Monkeypox for being gay?” “For God’s sake, monkeypox is not a gay disease. Learn more!” “I am so depressed right now. I know I shouldn’t, but these are the times that I wish I was not gay” “I am very concerned that we are going down the same pathway as the HIV/AIDS crisis of the 80s and 90s.”

^1^*N* does not add to 100% given that some posts contain more than one theme, and some were general mentions of mpox

^2^Posts were rephrased and altered

#### Changes in Sexual Behaviors and Social Activities

The US CDC recommends that at-risk individuals avoid or reduce behaviors that could increase exposure to mpox, including reducing skin to skin or face to face contact, limiting the number of sexual partners, and considering having sex with clothes [34]. A major theme of Reddit posts on mpox is the changes in sexual behaviors to mitigate the risk of potential mpox virus exposures among GBMSM during this outbreak. Many GBMSM on Reddit posted that they had stopped seeing casual sexual partners or being extremely cautious about the people they were intimate with. Some users said, “I have been watching and reading a lot of the posts and stories about monkeypox. As someone who used to hookup a lot, I think it’s time to take a break…” Similarly, the recent outbreak also caused disruptions for GBMSM to attend social activities, including social distancing from their friends and networks and attending community social venues, such as going to gay bars, clubs, and saunas (e.g., “I’ve made the decision that all my interactions with gay ppl will be socially distanced. I will not even hug or kiss my gay friends and will stop going to gay bars.”). Some GBMSM also explained the reasons for the changes in sexual behaviors. These include their perceived risks of exposure and infection. A small number of posts were also related to individuals who experienced mpox symptoms after receiving a positive test and followed social distancing procedures, “I got the Monkeypox... It doesn’t affect my health too much, but I do lose a ton of money on a holiday I planned overseas because of the social distancing measure. You have to quarantine for 21 days.”

#### Mpox Vaccine Attitude, Uptake, and Hesitancy

Another emerging theme of mpox-related posts on Reddit is mpox vaccine uptake, concerns, hesitancy, and attitude. Vaccination is an important tool to prevent the spread of the mpox virus, and sexually active GBMSM is among the priority populations to receive mpox vaccines [35]. In Reddit, many GBMSM expressed their attitudes towards mpox vaccination, including their desire to take the vaccine to protect themselves. Some also posted to inquire about vaccination availabilities and their eligibility to receive a vaccine, and among those who had taken the vaccine, some shared their experiences, such as having mild reactions, friendly interaction with the health providers, and their gratitude for receiving the protection (e.g., “Just get my frist dose of the monkeypox vaccine and it was such a positive experience. The staff were so nice and made me very comfortable. Go get vaxxed guys!”; “Just received my 2^nd^ dose and I am so glad to have it out the way”), while others also shared their side effects (“I got mine earlier this week. Very moderate reactions. Lots of nausea, coughing, and overall bleh feeling. But no fever. Arm is still a bit sore.”). Lastly, some Reddit users posted on Reddit to ask for their peers’ attitude on the mpox vaccine (e.g., “Have You Received The Monkeypox Vaccine? If you haven’t already, will you be getting it?”), while others seem hesitant about receiving the vaccine and ask for their peers’ attitudes towards monkeypox vaccination: “It seems like it is recommended to all men who have sex with men. Would you have it if it was available to you in your city?”.

#### Perceived and Experienced Stigma and Homophobia and Mental Distress

To date, most of the confirmed mpox cases outside of endemic countries in this outbreak are reported among GBMSM. However, the virus can affect anyone regardless of sexual orientation and gender identity [36]. Since the start of this outbreak, GBMSM have been stigmatized and blamed for spreading the virus. Message stereotyping and blaming GBMSM as the source and carrier of mpox have been rampant on social media. GBMSM on Reddit shared their experiences of perceived stigma and experienced stigma as gay or bisexual men. For example, some asked if the other users have witnessed increasing anti-gay sentiment related to mpox (“Anyone else seeing a rise in anti-gay sentiment since the monkeypox outbreak?”; “I told my parents about the mpox and they told me gays and gay sex are disgusting.”)

For many, these perceived and experienced stigma and discrimination had negatively impacted their mental well-being (e.g., “Feeling depressed because of the homophobia… But seeing all the homophobia from people who call monkeypox a gay disease and spread harmful misinformation is making me even more depressed”). Some individuals even consider the outbreak as a significant safety concern, “I am losing friends, and I can’t even go out because I live close to a very conservative state”; “I literally just came out last year, but it seems like I have to get back to the closet again for my safety”.

For some, the outbreak echoes the past and reminds them of the early years of the HIV/AIDS epidemic. Indeed, the first cases of HIV/AIDS in the 1980s were identified among gay men, which was quickly labeled the “gay disease”. This outbreak has disproportionately impacted the GBMSM, also labeled “gay pox”. Many GBMSM on Reddit discussed their thoughts on the parallels between the health crisis (e.g., “How the media’s homophobic and racist coverage of monkeypox mirrors the HIV/AIDS epidemic”, “The monkeypox crisis seems to have brought back a lot of the hysteria from the beginning of the AIDS crisis.”). Individuals also shared their concerns about the mpox outbreak becoming another HIV/AIDS epidemic, “I am very concerned that we are going down the same pathway as the HIV/AIDS crisis of the 80s and 90s.”; “many people believe that this is a new STI that mostly impacts gay men, or essentially a new HIV/AIDS crisis, which is definitely wrong”. Some also shared their thoughts on the lessons learned from the HIV/AIDS and encourage others to be cautious (e.g., “I have been through the HIV crisis in the 80s, here are some lessons we learned.”).

#### Online Information-Seeking and Mutual Aid and Support

During this unprecedented time, GBMSM used Reddit as a platform to seek and share mpox-related information. Such examples include asking for and sharing vaccination information (e.g., “Where in Austin can I get the monkeypox vaccine if I don’t have health insurance?”, “Below is a list where you can call up and get vaccinated, if you’re in a [high risk group] ... It could take up to a year to be contacted by the public health, so if you’re eligible, try and get yours asap.”). GBMSM asked questions about potential side effects of receiving an mpox vaccine (e.g., “What were your monkeypox Vaccine side effects like?”, “Anyone have a worse reaction after 2nd monkeypox vaccine?”). Individuals asked for advice from the other users to evaluate their risks and ways to mitigate mpox risk. For example, one asked, “What do you do to avoid monkeypox? It seems like there is not enough vaccine for everyone at risk. What are you doing to avoid the virus?”. Individuals also asked questions about their eligibility to receive mpox vaccines and if it is appropriate to take the vaccine even if they were not sexually active (e.g., “I want to get vaccinated, but I haven’t really been having sex lately. I wouldn’t feel right taking a vaccine from someone who may need it more than me. Should I just get it?”). GBMSM tended to describe their situations, including their relationship and sexual status, for the others to evaluate (e.g., “Should I get the monkeypox vaccine if my boyfriend and I are fully exclusive - I don’t particularly feel at any heightened risk more than any straight people?”).

GBMSM supported and participated in mutual aid on Reddit by advocating for safe behaviors and establishing a resource list. They shared resources as illustrated above and encouraged each other to raise awareness of the outbreak among their social networks (e.g., “Monkey Pox has been declared a global health emergency lately. Please be safe guys! Please spread awareness among your circles and friends”). They also suggested that vaccinated individuals should continue taking precautions and be careful (e.g., “It’s clear that one shot isn’t enough to give you full protection. Don’t be hasty and go hookup immediately after getting vaccinations. These things aren’t magic and they take time to become effective.”). Several posts were from individuals who tested positive for mpox, and they shared their experiences to help others with awareness: (“Hello everyone, I received some unfortunate news that I tested positive for Monkeypox right before my upcoming trip overseas. I wanted to share my experience to raise awareness of the symptoms and encourage everyone to take precautions to protect themselves. Additionally, I want to remind people that it’s possible to have a sexually transmitted disease (STD) and still receive negative test results, so it’s important to stay informed and seek medical attention if you suspect something is wrong. Although it may be difficult, try to stay positive and know that each case is different. I hope my story can help someone else in their own journey.”).

Finally, GBMSM on Reddit suggested that information and guidelines on protecting themselves were unclear (e.g., “Thankfully we know how to protect against HIV (condoms, PrEP and PEP). But information is very unclear about how to protect ourselves from Monkeypox”). Some also advocated establishing mpox-specific threads to inform the users and provide references: (“There should be a Monkeypox megathread … With the monkeypox causing a havoc with increasing social stigma, I think someone should start a ‘gaysovermonkeypox’ or ‘gaysovermpv’ just like they did with ‘gaysovercovid’”).

## Discussion

This current study examined the changes and themes in posting mpox-related content on a public social media platform, Reddit. Among several GBMSM-friendly subreddits, there was an overall increase in mpox-related posts over time since the outbreak in May 2022. In particular, we witnessed three upticks in mid-May, late July, and early August, which could correspond to the dates that the first case was reported in the USA, the WHO declared mpox a public health emergency, and the Department of Health and Human Services declared a public health emergency in the USA [3, 4]. This suggests that GBMSM utilize public social media platforms for information seeking and sharing during public health emergencies such as the mpox outbreak. The anonymous discussion forum may provide a safe space where GBMSM individuals could share their options openly and timely without the concerns of confidentiality or personal information exposure. Thematic analysis of the Reddit posts indicated that GBMSM employed various strategies to mitigate the potential exposure to the mpox virus. This includes reducing casual sexual partners and sexual behaviors, avoiding attending social venues, and taking mpox vaccines. These findings are in line with recent studies in the USA that GBMSM were taking actions to reduce their risk of acquiring or transmitting the virus [37, 38]. In a convenient sample of GBMSM recruited online, Delaney et al. found nearly half of the participants reported reducing their sexual partners and one-time sexual encounters. More than 40% reduced attendance to social events and sex venues due to the mpox outbreak [37]. These findings suggest that GBMSM are following the guidelines and recommendations of public health to protect themselves during this unprecedented time [39]. However, some were unclear about the guideline or could not evaluate their risks independently, suggesting the urgent need to deliver tailored and precise prevention messages to diverse GBMSM to meet their specific needs.

We found that GBMSM posted on Reddit to discuss a wide range of topics related to the mpox vaccine. To date, vaccination is still among the primary prevention strategies for mpox, and data suggested that the mpox vaccine is at least 85% effective in preventing infection [35, 40]. We found that many GBMSM were actively posting and seeking information about vaccination in their respective locations, indicating their desire and confidence in mpox vaccination in preventing themselves from acquiring the virus. Some GBMSM said they would not feel safe to have sexual behaviors until they were vaccinated, suggesting their high vaccine confidence. However, some also expressed their concerns about getting vaccinated for various reasons. First, GBMSM were not clear about their eligibility, especially given the frequently changed guidelines and criteria. For others, such hesitancy came from their concerns about vaccine side effects and effectiveness. Public health strategies and interventions to increase mpox vaccine uptake and testing uptake among GBMSM must address the historical medical mistrust and promote vaccine education. A recent report from the CDC suggested that mpox virus may cause breakthrough infections among vaccinated individuals, reflecting the ongoing community transmission [41] despite the overall decline in new cases since peaking in August 2022. Therefore, there is still a need to continue promoting mpox testing and screening and vaccination uptake and encouraging individuals to mitigate mpox exposures.

One potential strategy is to disseminate vaccine information and knowledge through popular opinion leaders (POL). Previous research and practice suggest that the POL model was highly acceptable among the GBMSM for HIV prevention and other sexual health promotions [42, 43]. In recent years, the POL model has been adapted to the Internet- and social media-based intervention and is found favorable to promote HIV prevention utilizations such as HIV testing [44, 45]. In this analysis, we found that some GBMSM posted on Reddit about their experiences of taking the vaccine and interactions with healthcare providers. For many, they encouraged each other to receive the vaccine as soon as it was available. This suggests an opportunity to engage community-trusted popular opinion leaders and social influencers to disseminate accurate public health and mpox vaccine information on social media to overcome the challenges in achieving equitable vaccine access and distribution. This is particularly important given that vaccine disparities are already emerging among the most vulnerable subgroups, including racial and ethnic minority GBMSM [37]. There are several ways such online platforms can be utilized to promote mpox vaccinations among the targeted populations, especially using the POL model. For example, public health official could consider creating informative and engaging content that highlight the importance of vaccination and available resources among targeted subreddits to generate conversations and discussions among key users. Public health officials can also collaborate with social media influencers or other POLs to help spread the messages and increase awareness among their followers and on these platforms. It is also critical for the authorities to monitor and respond to the feedback from the community members, such as GBMSM on Reddit to address their concerns, hesitancy, and questions regarding vaccination and testing uptake.

Lastly, the recent mpox outbreak has impacted GBMSM’s mental well-being. Some GBMSM posted on Reddit that they were anxious about the outbreak and their perceived risk of acquiring the virus. Some suggested that they had already experienced mpox-related stigma and discrimination as a member of the GBMSM. Others also expressed the parallel feeling that echoes back to the start of the HIV/AIDS epidemic, when HIV/AIDS was labeled a “gay disease”. Since the beginning of the outbreak, there has been massive social media coverage blaming GBMSM for spreading the virus [46]. This process could worsen the mental health status and create additional psychological stressors already faced by GBMSM, including those living with HIV, which the COVID-19 pandemic has also exacerbated [47–52]. This is particularly insidious given that it could deter GBMSM from seeking healthcare services. Public health programs and strategies to combat the spread of the virus and outbreak must be designed to reduce potential stigma and discrimination and integrate mental health services.

This study has some limitations. First, we only analyzed data from publicly available posts on Reddit. The generalizability of the study findings to those who did not post on Reddit or non-Reddit users is unknown. We only extracted data from several subreddit, given the popularity among GBMSM. Therefore, it is possible that the posts under other subreddits could be different. Another limitation is that it is impossible to verify if the posts were made by GBMSM users given that everyone can access and post on Reddit. Next, we cannot provide disaggregated data and information on specific user subgroups due to the limited information available. This includes the user’s geolocation information to distinguish the differences in mpox-related behaviors and outcomes across geographic regions and countries. This could be particularly informative in examining the differences in changes in sexual behaviors and attitudes towards mpox vaccines. Lastly, we did not assess if any of the themes had changed over time (May–August vs. August–October). Future studies should examine these changes longitudinally, especially given the rapid changes in vaccine eligibility and behavioral guidelines.

## Conclusion

We conducted a thematic analysis using publicly available data on a social media platform, Reddit. The results suggest that GBMSM had changed their sexual behaviors and practices to mitigate potential mpox virus exposure. Vaccine hesitancy was also observed through the content analysis. Future strategies to promote mpox vaccine uptake should consider engaging popular opinion leaders and utilizing social media as a platform for intervention delivery.

## Data Availability

All data were publicly available on http://reddit.com.
